# The importance of neutral over niche processes in structuring Ediacaran early animal communities

**DOI:** 10.1111/ele.13383

**Published:** 2019-09-12

**Authors:** Emily G. Mitchell, Simon Harris, Charlotte G. Kenchington, Philip Vixseboxse, Lucy Roberts, Catherine Clark, Alexandra Dennis, Alexander G. Liu, Philip R. Wilby

**Affiliations:** ^1^ Department of Earth Sciences University of Cambridge Downing Street Cambridge CB2 3EQ UK; ^2^ British Geological Survey Nicker Hill Keyworth, Nottingham NG12 5GG UK; ^3^ School of Earth Sciences University of Bristol Wills Memorial Building, Queens Road Bristol BS8 1RJ UK; ^4^ Department of Zoology University of Cambridge Downing Street Cambridge CB2 3EJ UK; ^5^ School of Geography, Geology & the Environment University of Leicester University Road Leicester LE1 7RH UK

**Keywords:** Ecology, Ediacaran, neutral theory, paleoecology, paleontology, spatial point process analysis

## Abstract

The relative influence of niche vs. neutral processes in ecosystem dynamics is an on‐going debate, but the extent to which they structured the earliest animal communities is unknown. Some of the oldest known metazoan‐dominated paleocommunities occur in Ediacaran age (~ 565 million years old) strata in Newfoundland, Canada and Charnwood Forest, UK. These comprise large and diverse populations of sessile organisms that are amenable to spatial point process analyses, enabling inference of the most likely underlying niche or neutral processes governing community structure. We mapped seven Ediacaran paleocommunities using LiDAR, photogrammetry and a laser line probe. We found that neutral processes dominate these paleocommunities, with niche processes exerting limited influence, in contrast with the niche‐dominated dynamics of modern marine ecosystems. The dominance of neutral processes suggests that early metazoan diversification may not have been driven by systematic adaptations to the local environment, but instead may have resulted from stochastic demographic differences.

## Introduction

Two opposing theories lie at the heart of the debate regarding the fundamental mechanisms that govern ecosystem structure and biodiversity: niche and neutral. Niche theory is a central tenet of classical ecological theory, whereby species avoid competitive exclusion by occupying different niches within the ecosystem (MacArthur 1984). Smaller niche overlaps result in less competition between taxa, permitting numerous taxa to co‐exist in an area without excluding each other. Species are able to coexist because they have different requirements. Niche models describe selection‐dominated ecosystems, whereby species dynamics operate deterministically as a series of interspecific interactions, which act as stabilising mechanisms for the ecosystem (Adler *et al.* 2007). In contrast, for neutral processes, species *similarities* drive high diversity rather than species *differences*, enabling co‐existence (Hubbell 2001). Within neutral models, species fitness is similar across a community, and so different taxa can co‐exist because no single taxon has a substantial competitive advantage over any other. Despite this seemingly unrealistic assumption, neutral theories have been able to accurately reproduce certain species–area distributions (Hubbell 2001) and beta diversity patterns (Condit *et al.* 2002; Chisholm & Pacala 2010), sometimes better than niche theories (Rosindell *et al.* 2011). In recent years, unified or continuous theories have emerged, whereby niche and neutral processes are combined to enable species coexistence (Gravel *et al.* 2006; Comdit *et al.* 2007). In these combined models, species can exhibit strong differences and strong stabilisations (niche‐type), or similar fitness and weak stabilisations (neutral‐type), with the classic niche and neutral models forming the extreme end‐members of such continuum models.

The evolution of macroscopic metazoans was coupled with a transformation in ecosystem dynamics, with paleocommunities emerging from pre‐Ediacaran microbial populations with assumed simple community structure (Butterfield 2007), via late Ediacaran (~ 571–540 Ma) paleocommunities that exhibited both simple and complex community structures (Darroch *et al.* 2018), into Cambrian ‘modern’ metazoan ecosystems with comparable ecosystem structures to the present day (Dunne *et al.* 2008). In order to investigate how niche and neutral processes contributed to community dynamics in deep time, we focus on some of the oldest macroscopic paleocommunities that are currently known: those comprising the Avalonian Assemblage of the Ediacaran macrobiota (Waggoner 2003; Boag *et al.* 2016).

**Figure 1:**
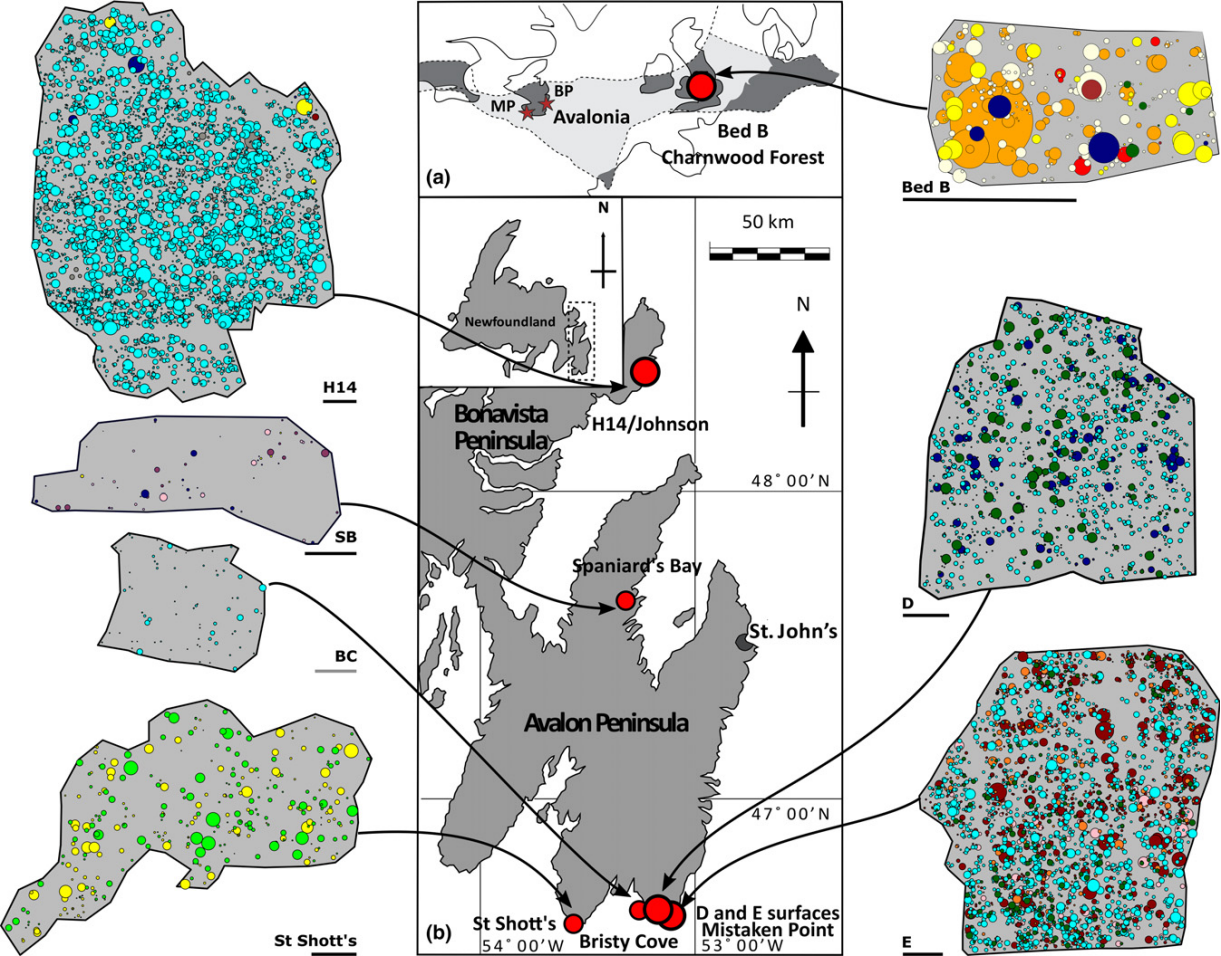
Locality map of study sites, showing: (a) the relative palaeogeographical locations of sites relative to the micro‐continent of Avalonia (grey shading), highlighting the Mistaken Point Ecological Reserve (MP) and the Bonavista Peninsula (BP) in Newfoundland, and Charnwood Forest, UK. (b) the Newfoundland sites of the ‘D’, ‘E’ and Bristy Cove (X‐Ray) surfaces, all in the Mistaken Point Ecological Reserve; the St. Shott’s (Sword Point) surface; the Spaniard’s Bay surface; and the H14/Johnson surface, Bonavista Peninsula (modified from Liu 2016). Associated spatial maps for each locality show the positions of the fossil specimens, where the size of the circle indicates the inferred *in vivo* vertical height of the organism. Black scale bar = 1 m, grey scale bar = 0.1 m. Different colours indicate different taxa as follows: *Thectardis* navy; *Fractofusus* light blue; *Charnia* bright yellow; *Charniodiscus* dark red; *Aspidella* light green; *Bradgatia* dark green; Feather Dusters light orange; *Primocandelabrum* dark orange; *Trepassia* dark purple; *Beothukis* bright pink; *Pectinifrons* dark blue; ‘Brushes’ brown; *Avalofractus* dark blue (Spaniard’s Bay only); *Hylaecullulus* light yellow.

The Avalonian Assemblage is known primarily from Newfoundland, Canada and Leicestershire, UK (Fig. 1) and is comprised entirely of soft‐bodied sessile organisms that are preserved *in situ* beneath volcanogenic/volcaniclastic event beds. These event beds have deep‐water depositional settings (Wood *et al.* 2003; Narbonne 2005) that are dated to ~ 571–560 Ma (Noble *et al.* 2015; Pu *et al.* 2016), and are considered to represent early animals (Budd & Jensen 2017; Dunn *et al.* 2018; Wood *et al*. 2019). As such, bedding plane surfaces are interpreted to preserve near‐complete census paleocommunities (Clapham *et al.* 2003; Wood *et al.* 2003), although the impact of modern erosion needs to be considered (Matthews *et al.* 2017; cf. Mitchell *et al.* 2015). Unlike hard parts, which can accumulate for thousands of years (Kidwell & Bosence 1991; Kowalewski & Bambach 2008), there was no potential for these soft‐bodied Ediacaran organisms to accumulate over such a large temporal extent. Avalonian ecosystems pre‐date macro‐predation and vertical burrowing, and so also remained undisturbed post‐mortem (Liu *et al.* 2011; Wilby *et al.* 2015; Mitchell & Butterfield 2018). Consequently, the size and position of each specimen can be considered an accurate record of the organism’s life history, including its dispersal/reproduction (Seidler & Plotkin 2006), and the habitat (Wiegand *et al.* 2006) and community interactions it was subject to (Getzin *et al.* 2006; Lingua *et al.* 2008; Getzin *et al.* 2008). Consideration of these fossil assemblages as census communities permits their investigation using the Spatial Point Process Analyses (SPPA) that are used in modern ecological studies (Illian *et al.* 2008; Wiegand & Moloney 2013). While interpreting processes from these spatial point patterns is imprecise (Law *et al.* 2009; McIntire and Fajardo, 2009), the use of several complementary SPPA, combined with the fitting of multiple different types of models, enables the most‐likely underlying processes to be inferred (Levin 1995; Waagepetersen and Guan 2009; Wiegand & Moloney 2013; Jalilian *et al.* 2013, McFadden *et al.* 2019).

SPPA can be used to investigate the underlying processes of community assembly because different types of spatial point models can be classified as either niche or neutral models (Harms *et al.* 2000; Seidler & Plotkin 2006; Lin *et al.* 2011; McFadden *et al.* 2019). Neutral processes are identified where intraspecific (univariate) distributions exhibit complete spatial randomness (CSR), or where dispersal processes acted independent of local environment (i.e. habitat heterogeneities; Diggle *et al.* 2005; Comita *et al.* 2007; Wiegand *et al.* 2007a, 2007b; Law *et al.* 2009). CSR indicates neutral processes because there are no biologically or ecologically significant intrinsic or extrinsic influences on the spatial distribution. Dispersal processes are also considered neutral since they describe cases where aggregations arise from propagules only travelling a limited distance, thus being unable to reach all suitable substrates regardless of underlying habitat heterogeneities or species requirements (Gunatilleke *et al.* 2006, Seider & Plotkin 2006). Niche processes are identified as intraspecific habitat associations, and/or non‐independent interspecific distributions (Lin *et al.* 2011; McFadden *et al.* 2019). Density‐dependent competition, as indicated by spatial segregation (Kenkel 1988), indicates a lack of sufficient resources, and is therefore a niche‐based process. For interspecific (bivariate) distributions, spatially independent patterns reflect neutral processes (McGill 2010), while non‐independent patterns result from niche processes such as resource competition, facilitation and/or habitat filtering (Wiegand *et al.* 2007b; Wiegand *et al.* 2009; McFadden *et al.* 2019).

## Methods

### Data collection and extraction

In this study, we assessed the univariate and bivariate spatial distributions of taxa from seven Avalonian bedding plane assemblages: the ‘D’, ‘E’, and Bristy Cove (X‐Ray) surfaces in the Mistaken Point Ecological Reserve (Clapham *et al.* 2003); the St. Shott’s surface at Sword Point; the H14 (Johnson) surface at Little Catalina (Hofmann *et al*. 2008); and the Spaniard’s Bay surface (Narbonne *et al*. 2009), all in Newfoundland, Canada; and Bed B in Charnwood Forest, UK (Wilby *et al*. 2011, Fig. 1 and Table 1). The spatial analyses require the mapping of large areas (up to 115 m^2^) at sufficient resolution to permit taxonomic identification of the specimens from the resulting digital data set. The method of mapping employed varied according to the nature of the bedding plane, in particular its dip and the fidelity of fossil preservation. We used LiDAR to scan all studied surfaces using a Faro Focus 330x, creating a 3D surface mesh (with a mean resolution of 1 mm) against which we referenced all subsequent data. The Spaniard’s Bay and Mistaken Point ‘D’ and ‘E’ surfaces were laser scanned using a Faro Scan Arm Laser Line Probe, generating surface meshes with a mean resolution of 0.050 mm. We scanned the areas in grids of 1 m × 1 m, incorporating control points common to the LiDAR scan to enable accurate alignment of the two data sets (performed using Geomagic 2015.1.1, Rock Hill, SC, USA). Fossil identification, position and dimensions (disc width, disc length, stem length, stem width, frond length and frond width) were digitized in Inkscape 0.92.3 on a 2D projection of the combined data set, resulting in a 2D vector map for each paleocommunity.

**Table 1:** Summary data for studied surfaces. Area is total area mapped. Total number of specimens includes non‐abundant taxa and taphomorphs. Density is of abundant taxa (i.e. those with > 30 individuals) only. Minimum size consistently preserved is the modal height of small specimens; specimens beneath this threshold are more prone to being overlooked and/or misidentified

Surface	Area mapped (m^2^)	Total number of specimens	Density (ind per m^2^)	Number of abundant (> 30) taxa	Minimum size of consistently preserved features (cm)
*Bed B*	115.16	761	6.61	3	1.20
*MP E*	85.42	2977	36.83	6	0.70
*MP D*	70.89	1402	22.29	3	1.20
*H14*	82.4	4235	52.55	1	1.50
*BC*	0.81	120	97.2	1	0.70
*St. Shott’s*	50.93	480	7.20	2	1.20
*Spaniard’s Bay* *	16.40	68	2.61	3	0.20

* Note, due to low specimen numbers, we included taxa with > 15 specimens as abundant on this surface (see SI discussion)

Fossil relief was not sufficiently high on the H14, Bristy Cove and St. Shott’s surfaces to permit accurate capture of all morphological details using the laser line probe. However, the fossils are readily discernible based on colour, allowing us to create a photomap using Agisoft Photoscan software v1.3.5 by combining a grid of overlapping images. Reference to the LiDAR scan allowed an orthomosaic with sub‐millimeter resolution to be generated, and the fossils were digitised as vectors as above.

Bed B has a dip of ~ 45° and so is not suitable for *in situ* high‐resolution scanning using our equipment. Instead, we generated a vector map of its fossils from Reflectance Transformation Images (RTIs) of casts of the surface, using RTI Builder from Cultural Heritage Imaging (Wilby *et al*. 2011, Kenchington *et al.* 2018; Mitchell *et al.* 2018). The LiDAR scan enabled the detection of mould deformation and, where necessary, was used to retrodeform the vector map.

We processed all complete vector maps using a custom script in Haskell (Jones 2003), which output specimen identification number, taxonomic identification and specimen dimensions for the subsequent spatial analyses.

### Taxonomic identification

Macrofossils present were assigned to one of twenty genera/morphogroups. These comprise thirteen taxonomically described groups and four forms that are widely recognised but remain to be formally described. Specimens that did not fit within these groups were assigned to one of two ‘bin’ groups (Shen *et al.* 2008), or to the taphomorph group (the decayed remains of already dead organisms, such as ivesheadiomorphs; Liu *et al.* 2011). Low abundance taxa (taken as < 30 specimens), organ taxa such as *Hiemalora*, and taphomorphs were excluded from analyses, leaving 12 abundant taxa: (1) *Avalofractus*, (2) *Beothukis*, (3) *Bradgatia*, (4) *Charnia*, (5) *Charniodiscus*, (6) Disc A (discs on the St. Shott’s surface with multiple concentric circles); (7) ‘Feather Dusters’, which includes *Plumeropriscum* and some *Primocandlebrum* specimens in Newfoundland; (8) *Fractofusus,* including both *F. andersoni* and *F*. *misrai*; (9) *Pectinifrons*, (10) *Primocandelabrum*, (11) *Thectardis* and (12) *Trepassia*.

Five of the identified taxa were not sufficiently abundant for use in our analyses: (13) *Arborea*, (14) “Brushes”, (15) *Hylaecullulus*, (16) “Ostrich Feathers” and (17) *Vinlandia*. The remaining specimens were classified as follows: (18) Ivesheadiomorphs (taphomorphs); (19) ‘Holdfast Discs’, which comprises of discoidal specimens of uncertain affinity, with or without associated stems, which lack sufficient detail to identify the parent taxon; and (20) ‘Other Species’, which describes rare forms that do not fall into any of the other groups (e.g. *Hapsidophyllas*). Three taxa (*Charniodiscus*, *Charnia*, *Bradgatia*) occurred abundantly on two bedding planes, and one (*Fractofusus*) on four bedding planes, enabling comparison among sites (cf. Mitchell & Butterfield 2018).

### Bias analyses

Tectonic deformation and differential erosion of the surfaces can distort spatial analyses (Narbonne 2005; Matthews *et al.* 2017). Hence, the impact of such deformation and erosion was tested and corrections were applied to surfaces if they significantly affected specimen density distributions (Fig. S1 and Table S1). We modelled differential erosion (cf. Mitchell *et al.* 2015) by fitting at least three heterogeneous Poisson models to the data, with the models dependent on *x* (parallel to strike), *y* (parallel to dip) and a point where erosion was considered most likely (e.g. nearest to the sea), and selected the best fit model by AIC score comparison. The St. Shott’s, Bed B and H14 surfaces all showed significant fossil density changes depending on these physical characters, implying that the observed surface specimen density has been significantly influenced by modern erosion. Such heterogeneous erosional processes were incorporated into subsequent analyses so that the underlying biological and ecological processes could be investigated. Tectonically distorted data were retrodeformed by restoring elongated holdfast discs to a circular outline (Wood *et al.* 2003).

### Spatial analyses

We performed initial data exploration, inhomogeneous Poisson modelling and segregation tests in R (R Core Team 2013) using the package spatstat (Berman 1986; Baddeley & Turner 2005). Programita was used to calculate distance measures and to perform aggregation model fitting (described in detail in Wiegand *et al.* 1999, 2006; Wiegand & Moloney 2004).

The univariate spatial distribution of each taxon on each bedding plane was described using the spatial summary statistic pair correlation functions (PCFs). PCFs describe how the (normalised) density of points (i.e. fossil specimens) at distance *r* from a typical point change as a function of distance (Illian *et al.* 2008; Wiegand & Moloney 2013; Baddeley et al. 2016). A PCF = 1 indicates complete spatial randomness (CSR); that is the point density is the same as the overall density. A PCF > 1 indicates aggregation, so a greater point density of points per area than the overall density; and PCF < 1 indicates segregation, so a smaller density of points per area than the overall density (Diggle 2003; Diggle *et al.* 2005; Illian *et al.* 2008). Univariate and bivariate PCFs were estimated from the population density using a bandwidth of 10 cm on all surfaces except Bristy Cove, where 1 cm bandwidth was used. To minimise noise, a smoothing was applied to the PCF that depended on specimen density. This smoothing was over three bandwidths for all bedding plane surfaces except Bristy Cove, which had a five bandwidth smoothing due to its much smaller area (< 1 m^2^). To test whether the PCF exhibited complete spatial randomness (CSR), 999 simulations were run for each univariate and bivariate distribution, with the 5% highest and lowest simulations used to define the simulation envelope (Levin 1992). CSR was modelled by a homogeneous Poisson model with a constant intensity function (homogeneous background) where the PCF = 1, and the fit of the fossil data to CSR was assessed using Diggle’s goodness‐of‐fit test (Diggle 2003; Diggle *et al.* 2005; Baddeley et al. 2016). Due to non‐independence of spatial data, Monte‐Carlo generated simulation envelopes cannot be interpreted as confidence intervals. If the observed data fell below the Monte‐Carlo simulations, the univariate or bivariate distribution was interpreted to be segregated, whereas the spatial distribution was interpreted to be aggregated if above the Monte‐Carlo simulations.

Where a taxon was not randomly distributed on a homogeneous background and was aggregated, its spatial distribution was fitted to a random model on a heterogeneous background (HP model, McFadden *et al.* 2019). Heterogeneous backgrounds are created by modifying the intensity function of the Poisson model to include a changing variable, which changes in proportion to the density of the taxon under consideration, being defined by a circle of radius R over which the density is averaged throughout the sample area. Heterogeneous Poisson models were formed using estimators within the range of 0.1 m < R < 1 m, with R corresponding to the best‐fit model used.

Thomas cluster models were fitted into the data where excursions outside the simulation envelopes for both homogeneous and heterogeneous Poisson models remained. Thomas cluster models are a type of parametric point process model that describe aggregation or a cluster process in which points are normally distributed about the centre point of the cluster – the parent point (Thomas 1949; Wiegand *et al.* 2007b). For a homogeneous Thomas cluster model, the parent points follow a homogeneous Poisson distribution, and for a homogeneous double cluster Thomas process (DTC), the parent points follow a Thomas cluster model that follows a homogeneous Poisson distribution. Inhomogeneous Thomas cluster models (ITC) result from realisations of a homogeneous Thomas cluster model that has been randomly thinned with the intensity function, thus resembling an additional action of habitat filtering (ITC) (Wiegand *et al.* 2007a; McFadden *et al.* 2019). Thomas cluster models were fit to the data as follows (c.f. Mitchell *et al.* 2015):

1. The PCF of the observed data and another distance measure, the L function (Thomas 1949; Besag 1974; Turkington & Harper 1979; Mahdi & Law 1987), were found. Both measures were calculated to ensure that the best‐fit model is not optimised towards only one distance measure, and thus encapsulate all spatial characteristics.
2. Best‐fit Thomas cluster processes (Wiegand *et al.* 2007b) were fitted to the two functions where PCF > 1. The best‐fit lines were not fitted to fluctuations around the random line of PCF = 1 in order to aid good fit about the actual aggregations, and to limit fitting of the model about random fluctuations. Programita used the minimal contrast method (Diggle 2003; Diggle *et al.* 2005) to find the best‐fit model.
3. Upon visual inspection, if the model did not describe the observed data well, the lines were refitted using just the PCF. If that fit was also poor, then only the L‐function was used.
4. 999 simulations of this model were generated, and the top and bottom 5% of simulations were used to create simulation envelopes. The fit was checked using the O‐ring statistic and the nearest neighbour function (Wiegand & Moloney 2004).
5. The *P*‐value from Diggle’s goodness‐of‐fit test (*p_d_*) was calculated over the model range using both the PCF summary function and the nearest neighbour function to check model fit. Very small‐scale segregations (under 2 cm) were not included in the model fitting since they likely represent the finite size of the specimens (Illian *et al.* 2008; Mitchell *et al.* 2015), and a lack of specimen overlap.
6. A univariate homogeneous Thomas cluster model was interpreted as the best model if there were no excursions outside the simulation envelope and the *p_d_* value was high.
7. If a univariate homogeneous Thomas cluster model was found not to be a good fit to the data, then an inhomogeneous Thomas cluster model (ITC) was fitted, whereby the background intensity varied according to heterogeneous Poisson models instead of a homogenous Poisson process (c.f. Fig. S2).
8. If two univariate homogeneous Thomas cluster models were found to be a good fit to the data at different spatial scales, then a double Thomas cluster model was fit to the data (DTC).

### Bivariate distributions

Spatial independence was tested using a toroidal shift model (Lotwick & Silverman 1982), whereby one pattern is fixed while the other is moved in its entirety. This analysis necessitated sub‐sampling of the mapped areas (to rectangular areas, Figs S3 and S4 and Table S2). Monte Carlo simulations of these toroidal shift models were run as detailed above and the bivariate distribution was taken to be non‐independent if there were excursions outside the simulation envelope.

### Assignment of niche or neutral processes to models

Categories of spatial models were assigned following McFadden *et al. *(2019). The null model (CSR) was tested via goodness‐of‐fit tests and Monte Carlo simulations, and if there were significant departures from CSR, the distributions were tested for HP, then TC and ITC. CSR and dispersal limitation patterns are neutral processes, with homogeneous Poisson models describing CSR processes, and Thomas cluster (TC) or Double Thomas cluster (DTC) models (Wiegand *et al.* 2007b) indicating dispersal limitation processes (Wiegand *et al.* 2007a; Lin *et al.* 2011). More generally, neutral processes can be indicated if there are significant aggregations that are independent of the environment (McFadden *et al.* 2019). Intraspecific habitat associations are best modelled by a heterogeneous Poisson model (HP) or, when combined with dispersal limitations, an inhomogeneous Thomas cluster model (ITC), (Lin *et al.* 2011; Harms *et al*. 2001; McFadden *et al.* 2019). Therefore, for univariate distributions, neutral processes are indicated by CSR, TC or DTC models, whereas niche processes are indicated by segregation, HP, and ITC models, as well as non‐independent bivariate distributions.

## Results

**Figure 2:**
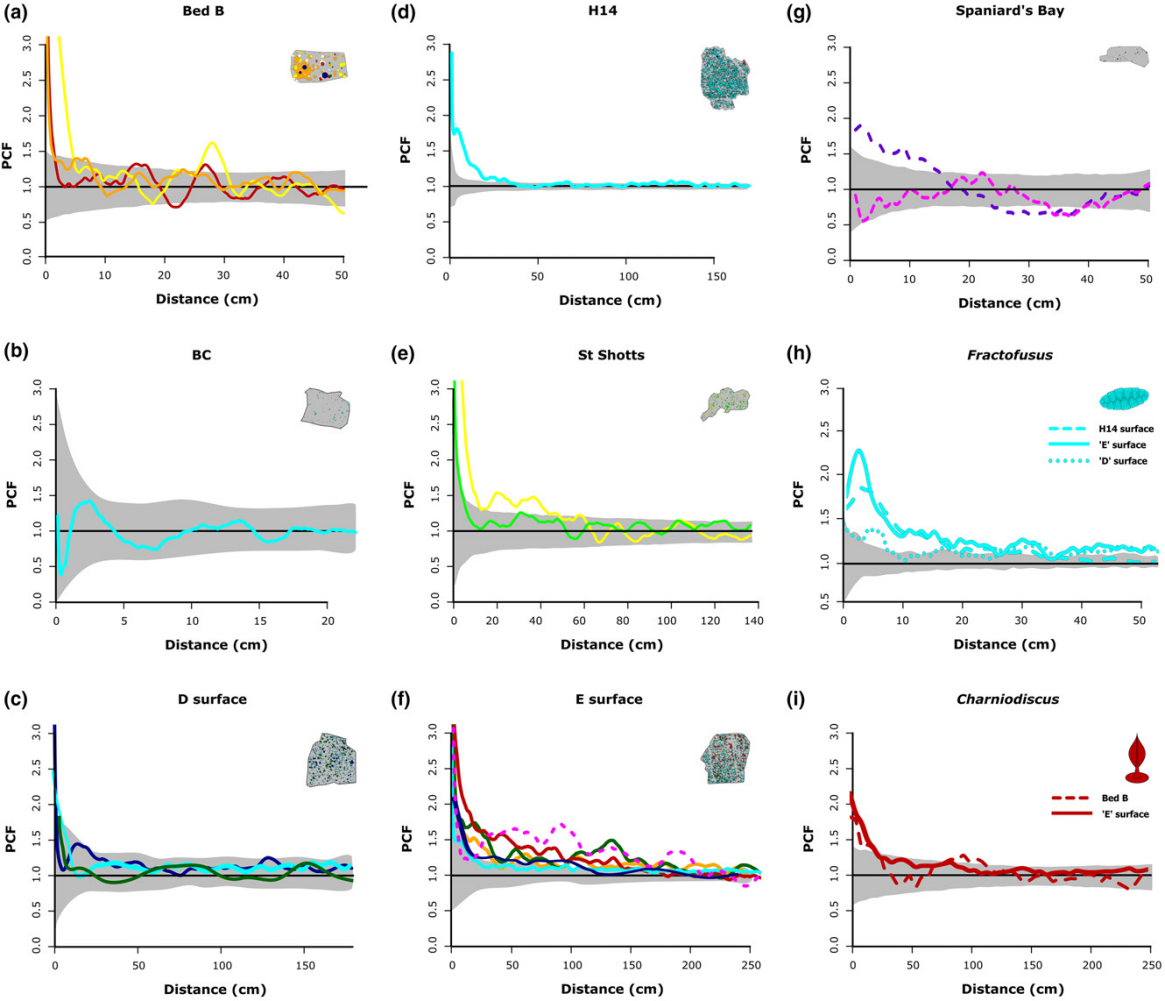
Univariate PCF analyses for the seven Ediacaran fossil surfaces (a–g) and for *Fractofusus* and *Charniodiscus* from multiple surfaces (h–i). (a) Bed B, Charnwood Forest, (b) Bristy Cove/X‐Ray surface, (c) ‘D’ surface, (d) H14/Johnson surface (e) St. Shott’s surface, (f) ‘E’ surface, and (g) Spaniard’s Bay surface. The univariate PCFs of (h) *Fractofusus* and (i) *Charniodiscus* demonstrating the consistency of their respective spatial distributions between localities. Where the best‐fit model for the distribution represents a niche process (Table 2), it is drawn as a dashed line. Models indicating neutral processes are drawn as solid lines. Black line represents the random model. The grey area is the simulation envelope for 999 Monte Carlo simulations of complete spatial randomness. The *x*‐axis is the inter‐point distance between organisms in metres. On the *y*‐axis PCF = 1 indicates complete spatial randomness (CSR), < 1 indicates segregation, and > 1 indicates aggregation. Different colours indicate different taxa as follows: *Thectardis* navy; *Fractofusus* light blue; *Charnia* bright yellow; *Charniodiscus* dark red; *Aspidella* light green; *Bradgatia* dark green; ‘Feather Duster’, light orange; *Primocandelabrum* dark orange; *Trepassia* dark purple; *Beothukis* bright pink; *Pectinifrons* dark blue; ‘Brushes’ brown; *Avalofractus* dark blue (Spaniard’s Bay surface only); *Hylaecullulus* light yellow. This figure shows that neutral processes (solid lines) dominated these Avalonian communities, and that *Fractofusus* and *Charniodiscus* show the same distributions across multiple surfaces.

The univariate and bivariate analyses combine to provide strong evidence that neutral processes dominated Avalonian Assemblage paleocommunities, with only limited niche‐based influence. Across the seven surfaces and the 18 univariate distributions examined, nine taxon distributions were assigned to CSR (Fig. 2 and Table 2). Most of the non‐CSR taxon distributions were best modelled by TC (or DTC). Only *Trepassia* on the Spaniard’s Bay surface was best modelled by an ITC model (Fig. 2g and Table 2). The only taxon to exhibit univariate spatial distribution with an HP best‐fit model was *Beothukis* on the ‘E’ and Spaniard’s Bay surfaces (Fig. 2g). These three instances of HP and ITC all had strong goodness‐of‐fit *p_d_* values compared to other models (Table 2). These results suggest that dispersal limitation processes structured the majority of spatial distributions of Avalonian communities.

**Table 2:** Summary table of univariate PCF analyses. For the inhomogeneous point processes (HP and ITC), the moving window radius is 0.5 m, using the same taxon density as the taxon being modelled

Surface	Taxon	n	Model fit			*p_d_ values PCF*				*p_d_ values NN*			
				100	Mean number in cluster	CSR	HP	TC	ITC	CSR	HP	TC	ITC
*Bed B*	*Charniodiscus*	48	2.906	0.001	0.001	0.001	0.002	0.892	0.005	0.190	0.117	0.596	0.553
	*Primocandelabrum*	106	21.47	0.124	0.124	0.124	0.002	0.647	0.137	0.251	0.268	0.981	0.255
	*Charnia*	69	12.027	0.294	0.294	0.294	0.266	0.972	0.272	0.020	0.020	0.923	0.071
*Bristy Cove*	*Fractofusus*	76	NA	0.523	0.523	0.523	0.182	0.173	0.183	0.410	0.396	0.368	0.389
*Mistaken Point D*	*Fractofusus*	1067	4.312	0.001	0.001	0.001	0.512	0.767	0.021	0.001	0.005	0.168	0.001
	*Pectinifrons*	108	NA	0.575	0.575	0.575	0.325	0.911	0.357	0.041	0.030	0.302	0.039
	*Bradgatia*	140	NA	0.664	0.664	0.664	0.425	0.466	0.397	0.544	0.482	0.505	0.512
*Mistaken Point E*	*Bradgatia*	34	NA	0.44	0.44	0.540	0.001	0.445	0.002	0.001	0.003	0.323	0.001
	*Charniodiscus*	326	2.133	0.01	0.01	0.001	0.132	0.451	0.142	0.001	0.001	0.074	0.001
	*Beothukis*	76	6.841	0.01	0.01	0.001	0.963	0.609	0.11	0.201	0.967	0.180	0.199
	Feather Dusters	272	5.616	0.01	0.01	0.001	0.019	0.282	0.183	0.002	0.001	0.206	0.001
	*Thectardis*	39	1.835	0.02	0.02	0.002	0.443	0.911	0.073	0.479	0.479	0.657	0.458
	*Fractofusus*	1137	11.842	0.01	0.01	0.001	0.031	0.325	0.35	0.001	0.001	0.971	0.001
*H14*	*Fractofusus*	3886	7.906	0.001	0.001	0.001	0.003	0.560	0.004	0.001	0.001	0.225	0.001
*St. Shott’s*	*Aspidella*	170	9.226	0.011	0.011	0.257	0.225	0.331	0.271	0.572	0.589	0.629	0.583
	*Charnia*	54	10.97	0.257	0.257	0.011	0.016	0.963	0.015	0.45	0.491	0.985	NA
*Spaniard’s Bay*	*Beothukis*	18	36.70	0.401	0.401	0.401	0.810	0.530	0.308	0.196	0.194	0.227	0.181
	*Trepassia*	33	7.148	0.005	0.005	0.005	0.022	0.980	0.850	0.576	0.570	0.572	0.574

Note.

*p_d_*_ _= 1 corresponds to a perfect fit of the model to the data, while *p_d_* = 0 corresponds to no fit. Where observed data did not fall outside CSR Monte‐Carlo simulation envelopes, no further analyses were performed, which is indicated by NA. σ: cluster radius, ρ: density of specimens, CSR: Complete spatial randomness, HP: Heterogeneous Poisson model, TC: Thomas cluster model and ITC: inhomogeneous Thomas cluster model. Mean number in cluster refers to the mean the mean number of individuals in a cluster estimated as λ/ρ. Note that if the cluster model is not a good fit, the mean number in cluster will not necessarily be appropriate. Note that three taxa (*Charniodiscus*, Feather Dusters and *Fractofusus*) on Mistaken Point E surface and *Fractofusus* on H14 are not well described by a best‐fit single Thomas cluster model because they are best described by a double Thomas cluster model (see Mitchell *et al.* 2015 for details).

A direct analysis of spatial scale could not be performed on our data because the palaeocommunities are limited in spatial scale, with the largest surfaces enabling spatial analyses of only up to 2.5 m. Instead, to gain some indication of whether taxa behave differently over broad spatial scales, we compared the univariate spatial distributions of individual taxa across different sites, thereby representing paleocommunities separated by broad spatial and temporal scales. *Bradgatia*, *Charnia*, *Charniodiscus* and *Fractofusus* all exhibit a consistent best‐fit model (CSR, TC, TC and TC/DTC, respectively) across all the surfaces on which they were observed (Fig. 2 and Table 2). Previous work has demonstrated that *Fractofusus* consistently shows the same spatial distributions across multiple surfaces (Mitchell *et al.* 2015) in different geological units (cf. H14 and ‘E’ surface, Fig. 2h). *Charniodiscus* also shows consistent spatial distributions (Fig. 2i), even between paleocommunities that are temporally and geographically separate (e.g. *Charniodiscus* from the ‘E’ surface and from Bed B). The consistency of univariate spatial distributions across multiple sites suggests that the small‐spatial‐scale ecological behaviour of these taxa is constant over broad spatial and temporal scales, providing further evidence of the importance of neutral processes to these communities.

**Figure 3:**
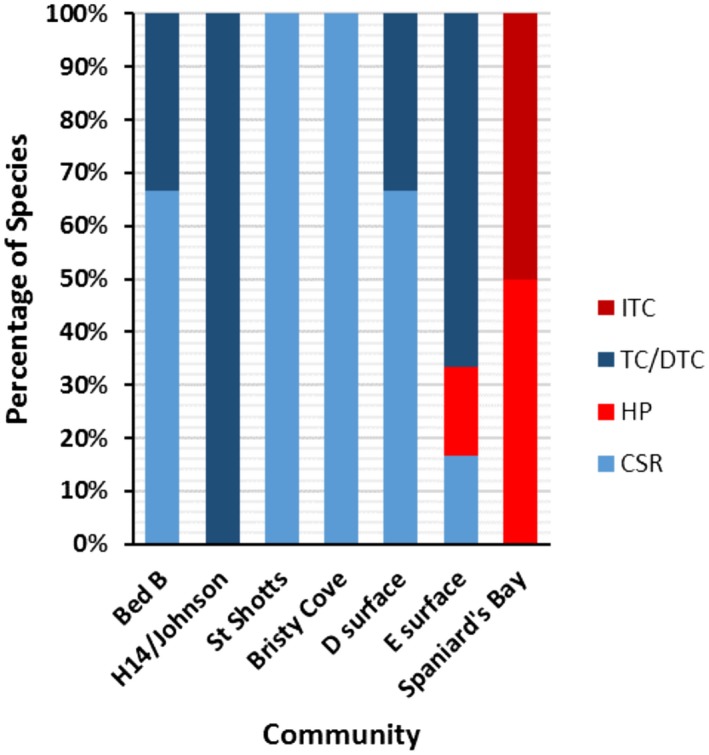
Proportion of best‐fit univariate models by surface. The percentage of taxa with univariate spatial distributions that are best described by CSR, HP, TC (or DTC) and ITC models. CSR and TC are considered neutral models and are shown in blue. HP and ITC are niche models, shown in red.

**Figure 4:**
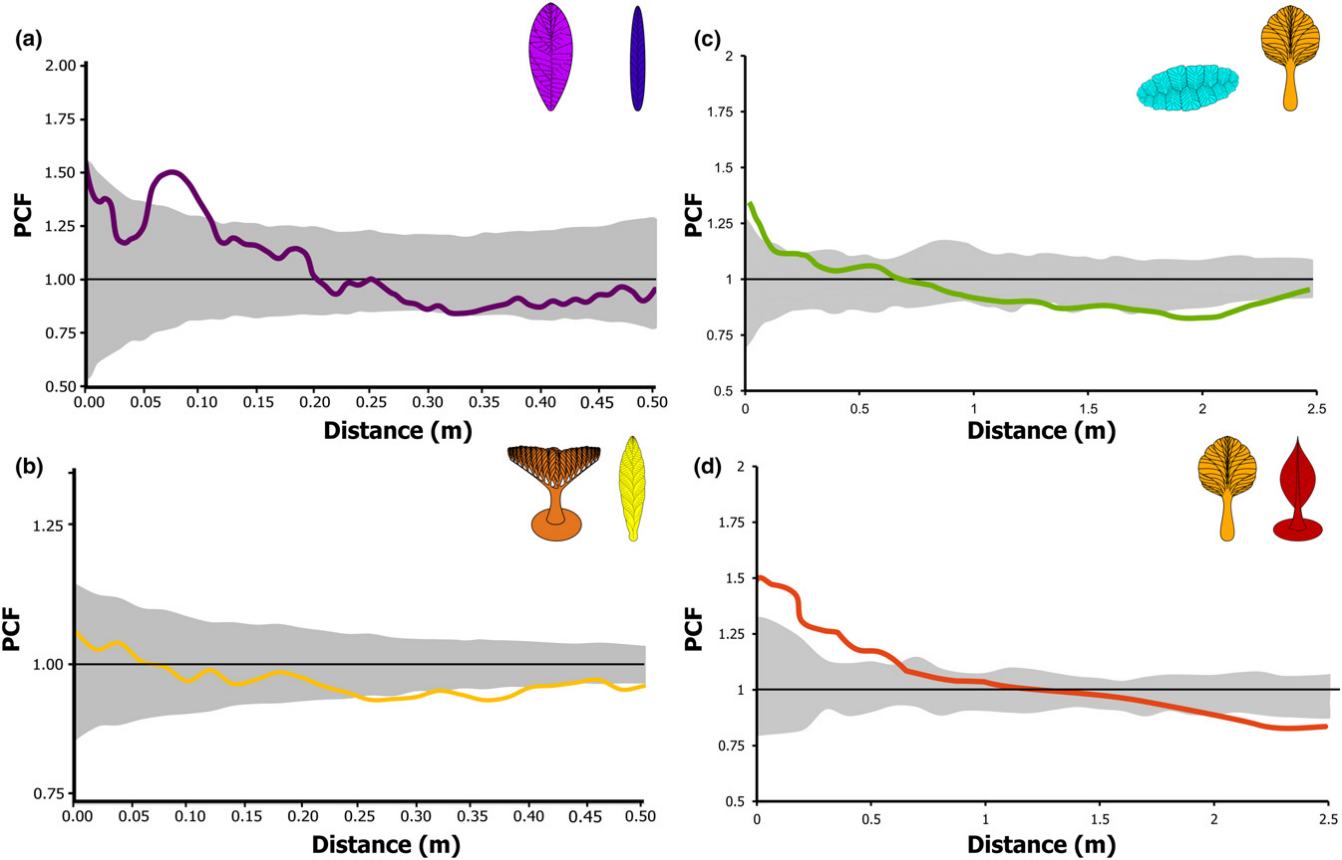
Bivariate PCF analyses for the taxa with non‐independent spatial distributions. The grey area is the simulation envelope of complete spatial randomness (CSR) for 999 Monte Carlo simulations CSR. The *x*‐axis is the interpoint distance between organisms in metres. On the *y*‐axis, PCF = 1 indicates CSR and is indicated by a black line, < 1 indicates segregation, and > 1 indicates aggregation. (a) *Trepassia* and *Beothukis* from Spaniard’s Bay. (b) *Charnia* and *Primocandelabrum* from Bed B, Charnwood Forest. (c) Feather Dusters and *Fractofusus,* and (d) Feather Dusters and *Charniodiscus* from Mistaken Point ‘E’ Surface. C and D reproduced from Mitchell and Butterfield (2018), with 99 Monte Carlo simulations of CSR.

Three of the five studied paleocommunities contained more than one abundant taxon, thus enabling bivariate distribution analyses (‘E’, Spaniard’s Bay and Bed B surfaces; Mitchell & Butterfield 2018; Mitchell & Kenchington 2018). Non‐independent bivariate distributions were rare, with only 4 out of a possible 24 bivariate distributions showing non‐independence, revealing that neutral processes also dominate bivariate taxa distributions (Fig. 4 and Table S3). Furthermore, even when present, these non‐independent bivariate distributions are weaker in PCF magnitude than univariate distributions (Figs 1 and 3). Significant bivariate aggregations increased specimen density by up to 56% (50% for Spaniard’s Bay *Beothukis* – *Trepassia* specimens; 34% for ‘E’ surface *Fractofusus* – Feather Dusters specimens; and 56% for ‘E’ surface Feather Dusters – *Charniodiscus* specimens respectively; Fig. 4a), while significant bivariate segregations reduced specimen density by up to 39% (39% for Spaniard’s Bay *Beothukis* – *Trepassia*, specimens; 7% for Bed B *Charnia* – *Primocandelabrum*; 10% for ‘E’ surface *Fractofusus* – Feather Dusters; and 10% for ‘E’ surface Feather Dusters – *Charniodiscus* respectively; Fig. 4). In contrast, the univariate dispersal‐generated aggregations increased taxon density by 180–300% (Fig. 2), indicating that bivariate processes had less impact on spatial distributions than univariate processes. Non‐independent bivariate distributions reflect interactions with local resources, so these spatial distributions suggest that influence from local resources was rare, and where present was relatively weak in magnitude (Fig. 4; cf. Mitchell & Butterfield 2018; Mitchell & Kenchington 2018).

## Discussion

This study provides strong evidence that neutral processes structured Avalonian Assemblage paleocommunities, with neutral best‐fit models dominating the univariate distributions, repetition of best‐fit univariate models across different paleocommunities, and weak and rare bivariate niche best‐fit models. These neutral‐process‐dominated community dynamics contrast with those observed in the modern marine realm, where neutral processes are typically rare across multiple spatial scales, from individual sites to regional scales (Dornelas *et al.* 2006; Connolly *et al.* 2014). This stark difference raises the question of whether Ediacaran early animal paleocommunities had fundamentally different community dynamics was to those of the present day. To our knowledge, the only other work investigating niche‐neutral influences in the geological record is from the Quaternary (2.58–0.01 Ma), where strong model and empirical support was found for environment‐led (niche) models of assembly (Jackson & Blois 2015), as in extant terrestrial systems. There are some important differences between Avalonian Assemblage paleocommunities and extant marine communities. Most notable is that Avalonian paleocommunities appear to differ from the majority of modern marine systems in the extent of their ecological maturity, generally preserving no more than three generations (Mitchell *et al.* 2015), though there is evidence for rare ‘survivors’ from earlier generations (Wilby *et al.* 2015), and for secondary community succession (Liu *et al.* 2012). These characteristics suggest that Avalonian communities are not always mature, and were perhaps curtailed by high frequency incursions of sediment, which acted to limit their maturity (Wilby *et al.* 2015). Recent simulations show that community dynamics in small populations living in fluctuating environments are dominated by neutral processes, implying a lack of small‐spatial scale environmental control on ecological dynamics in such systems (Fisher & Mehta 2014).

The relative influence of niche vs. neutral processes is affected by taxon dispersal ranges (Ron *et al.* 2018). Wide dispersal ranges increase the connectivity between populations, and so expand effective community size, with the net result of enhancing ecological selection (competition) and thereby increasing the relative importance of niche processes (Ron *et al.* 2018). The opposite is true when dispersal is limited, making such communities more likely to be dominated by neutral processes (Ron *et al.* 2018). The widespread distributions of certain Avalonian taxa (e.g. *Charnia*) provide evidence that these, at least, were capable of wide spatial dispersal (Darroch *et al.* 2013; Boag *et al.* 2016). The CSR and HP distributions of *Beothukis* and *Bradgatia* (Table 2) similarly suggest wide dispersal. In contrast, six of the seven studied paleocommunities were dominated by taxa which predominantly exhibit limited local dispersal (Fig 2a, h and i; Mitchell & Butterfield 2018, Mitchell & Kenchington 2018), with the PCF plots for *Fractofusus, Charniodiscus* and *Primocandelabrum* (Fig. 2a, h and i), suggesting typical dispersal ranges of < 20 cm. However, *Fractofusus* was also capable of a wide‐reaching waterborne propagule stage, although these only make a minor contribution to the population (Mitchell *et al.* 2015), and the global distribution of *Charniodiscus* suggests that it may have been similarly capable. The studied Ediacaran paleocommunities have comparatively small populations, experienced frequent disturbance events, and include many taxa with short dispersal ranges, so within this framework we would expect neutral processes to dominate. While the dominance of neutral processes within these paleocommunities differs substantively from the majority of the modern marine realm, the underlying dynamics are entirely consistent with models of assembly that include both niche and neutral processes, and are similar to those of modern communities subject to the same conditions. Thus, it is therefore likely that the fundamental mechanisms of metazoan community assembly were already in place in the Ediacaran Period, and so may have existed unchanged for ~ 570 million years.

### Resource limitation and spatial scales

The relative importance of niche and neutral processes to community assembly generally changes with spatial scale in modern communities (Garzon‐Lopez *et al*. 2014; Chase 2014). SPPA analyses of modern deep‐sea benthic systems have yet to be performed over a range of spatial scales, so direct comparison with the Avalonian Assemblage palaeocommunities is not possible. Instead, we rely on analyses of terrestrial forests. In extant forests, larger spatial scales (~ 1000 m^2^) show strong habitat associations, whereas smaller spatial scales (~ 1 m^2^) show stronger neutral processes (Garzon‐Lopez *et al*. 2014). This pattern is unlikely to be repeated in extant deep‐sea communities because limited resources in such environments lead to clear niche‐processes (spatial segregation) at the small (~ 1 m^2^) spatial scales (Gage & Tyler 1991; Tecchio *et al.* 2011). Hence, it is unlikely that spatial scale alone can explain the dominance of neutral processes in Ediacaran communities at the metre scale.

In contrast to modern deep‐sea benthic communities, which are highly resource‐limited (Gage & Tyler 1991; Tecchio *et al.* 2011), the Avalonian communities do not appear to have been restricted by lack of resources, as demonstrated by the rarity of competition for resources (Mitchell & Kenchington 2018) and dominance of neutral processes. We would expect to see evidence of niche processes at the spatial scales presented here because the environment of the Ediacaran seafloor may have differed at the meter scale because microbial mats (which are interpreted to have covered the Ediacaran seafloor) can show variability in composition and structure even over distances of just a few centimetres (e.g. McKay *et al.* 2012). Even if Ediacaran substrates were homogeneous, sub meter‐scale environmental filtering is still possible: the highly limited resources of the modern deep‐sea environment lead to spatial segregation at sub‐meter scale (Tecchio *et al.* 2011). As such, the lack of evidence for environmental influence in these communities suggests that the dynamics found in these communities are characterised by environments that are not resource limited, enhancing the importance of stochastic processes for Avalonian community assembly.

### Evolutionary context

In a similar manner to ecological processes, evolutionary processes can be categorised as niche (selection) or neutral (drift) processes (Chave 2004). Selection (niche) processes are considered deterministic because external factors, such as limited resources, lead to competition in a predictable way: given a set of initial conditions, the organisms/communities will always respond to these conditions (environment) in the same way (Chave 2004). By contrast, drift (neutral) processes are considered stochastic because they result from random fluctuations in population demography, so given a set of the same initial conditions, different populations/communities may emerge. Hence, the observed dominance of neutral ecological processes in the Ediacaran Avalonian paleocommunities establishes that they are inherently stochastic/probabilistic, with the possible implication that early metazoan diversification was not a systematic adaption to optimise survival under prevailing environmental conditions (which would be niche processes, and so deterministic). Instead, the existence of Avalonian organisms under a stochastic regime favours a scenario in which diversification was driven by demographic differences resulting from random within‐population differences. If this hypothesis is correct and early metazoan evolution was stochastic, then this stochasticity may help to explain why neutral models of evolution can reproduce substantial macro‐evolutionary trends such as the Cambrian Explosion (cf. Budd & Mann 2018), despite the known importance of niche processes in shaping evolution more broadly (e.g. Hutchinson 1957).

## Conclusions

We have shown that paleocommunities of early macroscopic metazoans were overwhelmingly dominated by neutral ecological processes, with only limited and weak evidence for niche processes. Our results strongly contrast with modern marine systems. Since the studied Ediacaran paleocommunities have traits (short dispersal ranges, small populations and frequent disturbances) that are associated with extant communities governed by neutral processes, our results suggest that the fundamental mechanisms of community assembly may have been in place since the early stages of metazoan evolution. The dominance of neutral processes in these paleocommunities suggests that systematic adaptation of the Ediacaran organisms to their local environment may not have been the underlying driver of early metazoan diversification. Instead, late Neoproterozoic metazoan diversification may have resulted from stochastic demographic differences, with only limited environmental influence.

## Conflict of Interest

The authors declare no competing interests.

## Authorship

EGM conceived the project, designed the research, ran the analyses and wrote the first draft of the paper. SH developed the data post‐processing protocol. SH and EGM processed field data. All authors contributed to field data collection and were involved in writing the final manuscript.

## Data Availability

The data supporting the results are archived in Figshare in https://doi.org/10.6084/m9.figshare.9576863. Due to geoconservation concerns, only a subset of data from Bed B will be made publicly available, with the full set available to researchers on request from the corresponding author.
