# Supplementary material for: The importance of neutral over niche processes in structuring Ediacaran early animal communities
Source: Ecol Lett. 2019 Sep 12;22(12):2028–38. doi: 10.1111/ele.13383 (PMC6899650; doi:10.1111/ele.13383)
Supplement: Supplementary file 1 [file ELE-22-2028-s001.docx]

Supplementary Information


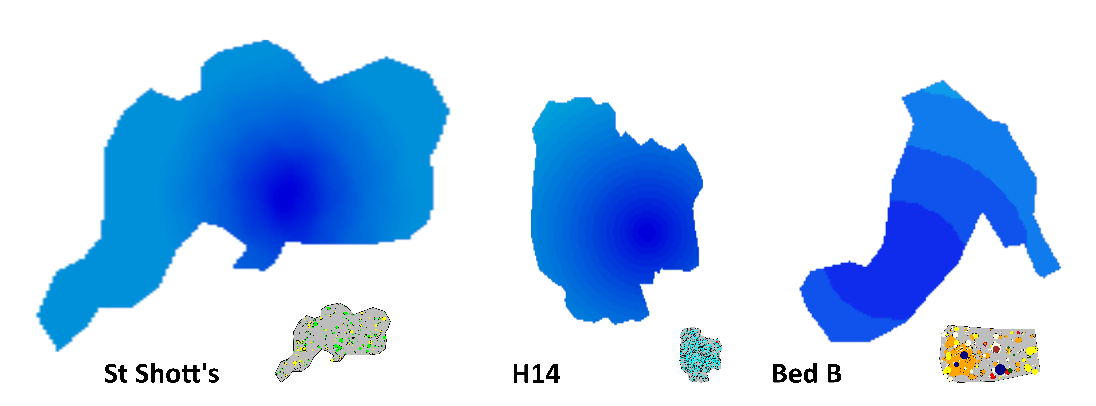


**Figure S1:** Model density maps of surfaces showing modelled erosion biases. For each surface darker colors indicate higher modelled fossil density, and therefore lower presumed erosional rate, normalised for the density on each surface. These models will not cover all erosional differences, just the ones that significant impact density. So while it is likely that the freshly exposed fossils have great preservation detail, the less-freshly exposed ones are still there, but will less detail. Note that Bed B has a coarser pattern due to a relatively lower fossil density difference across the surface and that the full spatial map is not provided due to concerns about fossil theft.

| Surface | x | y | √[(x-x_1_)^2^ + (y-y_1_)^2^ ] | x_1_ | y_1_ |
| --- | --- | --- | --- | --- | --- |
| *Bed B* | 3.26 | **6.94** | 1.54 | 89 | 112 |
| *Bristy Cove* | -1.93 | -1.93 | -1.77 | 84 | 91 |
| *Mistaken Point D* | -3.37 | -12.29 | -12.51 | 52 | 939 |
| *Mistaken Point E* | -75.64 | -5.06 | -72.29 | 309 | 283 |
| *H14* | 43.26 | 37.69 | **87.34** | 98 | 70 |
| *St. Shott’s* | -0.36 | 14.17 | **23.82** | 93 | 113 |
| *Spaniard’s Bay* | 0.14 | -1.96 | -1.78 | 294 | 143 |

**Table S1: ΔAIC values for density models used to investigate erosional biases.** *x* is parallel to strike, *y* parallel to dip, *√[(x-x_1_)^2^ + (y-y_1_)^2^ ]* is the distance from the point of least erosion, and x_1_ and y_1_ are the co-ordinates of that point. These ΔAIC were used to determine the best-fit models. ΔAIC > 0 indicates that the model has a better fit to the data than completely spatially random model. Units are the centimetre co-ordinates of the spatial maps.


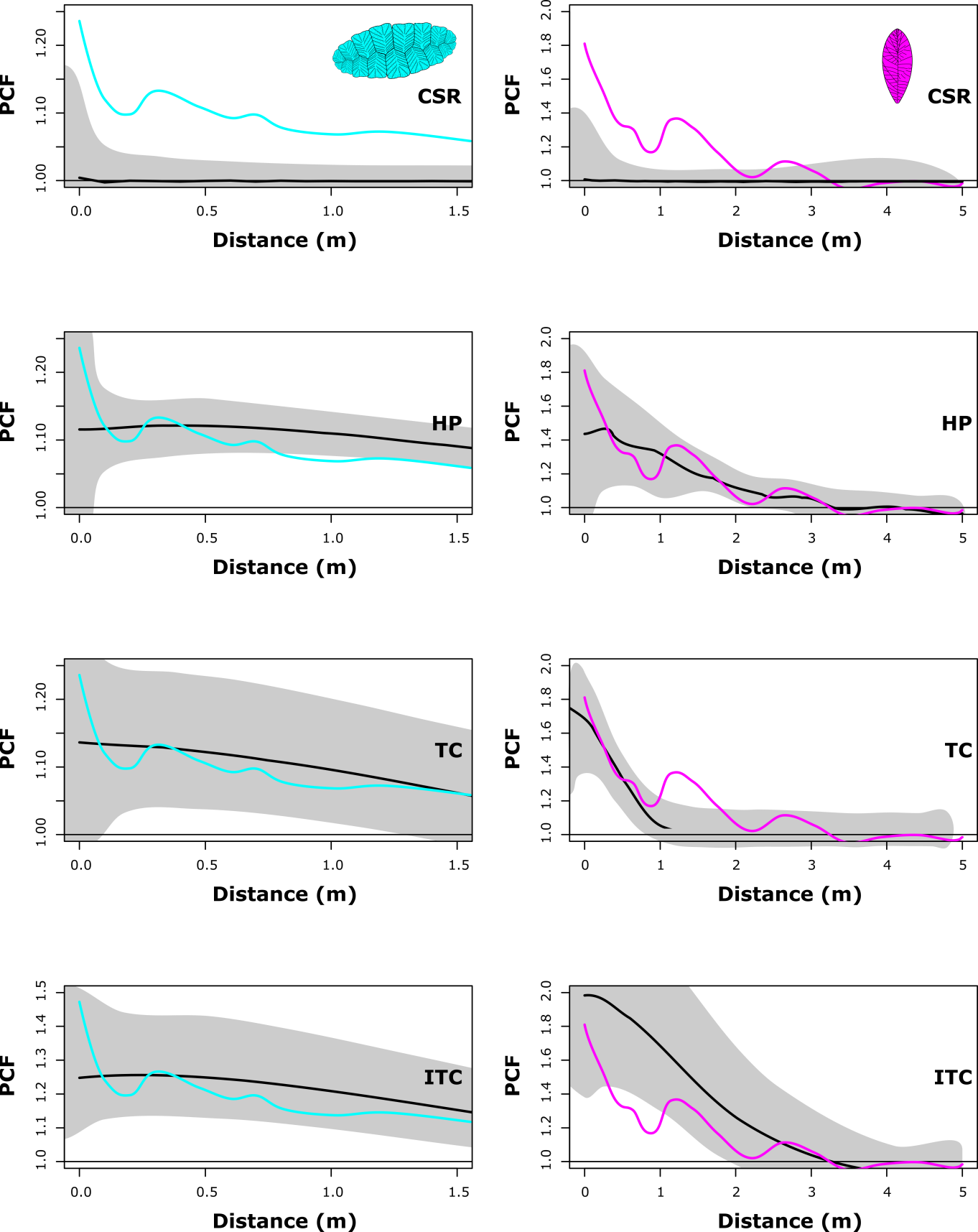


**Figure S2.** Univariate PCF analyses for *Fractofusus* on the ‘D’ surface (left) and *Beothukis* on the ‘E’ surface (right) for four different spatial models: CSR, HP, DTC, ITC (see text for model discussion). The model lines are black, the grey area represents the simulation envelope of 999 Monte Carlo simulations and the coloured lines are the observed spatial distributions. For *Fractofusus* (left) the best-fit model is TC, whereas for *Beothukis* (right) it is HP because they follow the model best as evidenced by the Monte-Carlo simulations and p_d_ value (Table S3).

**
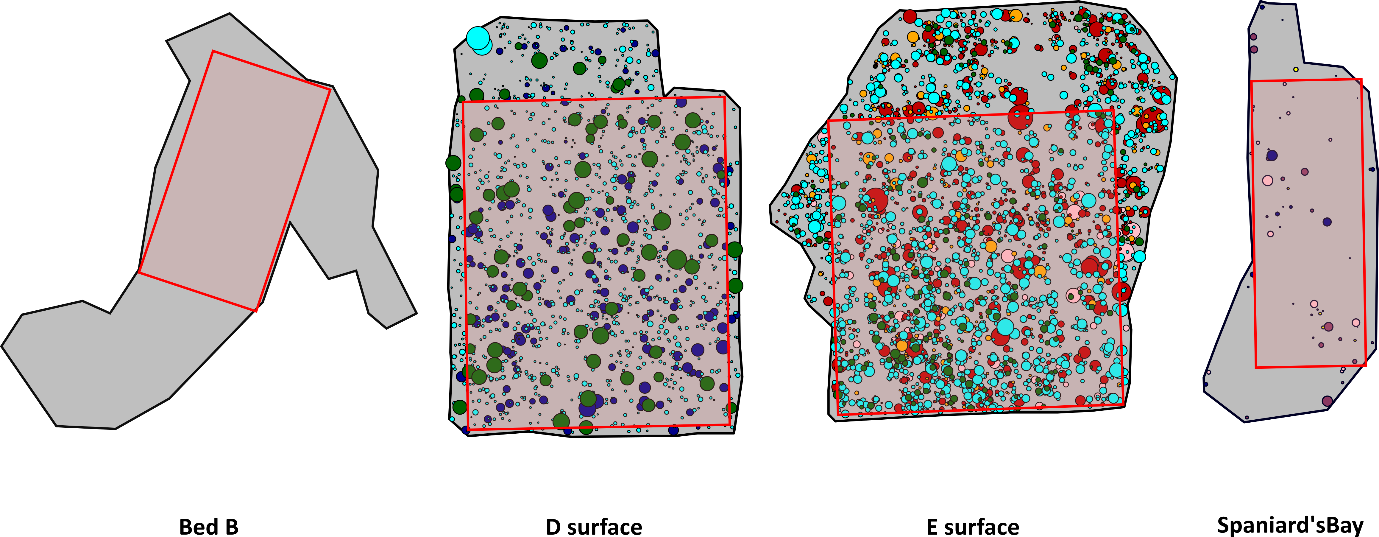
**

**Figure S3.** The cropped area for the Toroidal shift analyses for the four surfaces with non-independent bivariate distributions. Cropped area used for analyses is given by the red shaded rectangle. Specimen positions are not given for Bed B due to geoconservation concerns.

| **Surface** | **Taxon 1** | **Taxon 2** | **PCF *p_d_*** | **NN *p_d_*** |
| --- | --- | --- | --- | --- |
| **Bed B** | *Charnia* | *Primocandelabrum* | 0.079 | 0.002 |
| **Mistaken Point E** | Feather Dusters | *Fractofusus* | 0.051 | 0.285 |
| **Mistaken Point E** | *Fractofusus* | Feather Dusters | 0.040 | 0.284 |
| **Spaniard’s Bay** | *Beothukis* | *Trepassia* | 0.050 | 0.005 |

**Table S2.** Summary Table of Torodial Shift bivariate analyses. *p_d_* = 1 corresponds to a perfect fit of the null model to the data, so indicates independence between the two taxa, while *p_d_* = 0 corresponds to no fit, so non-independence between taxa.


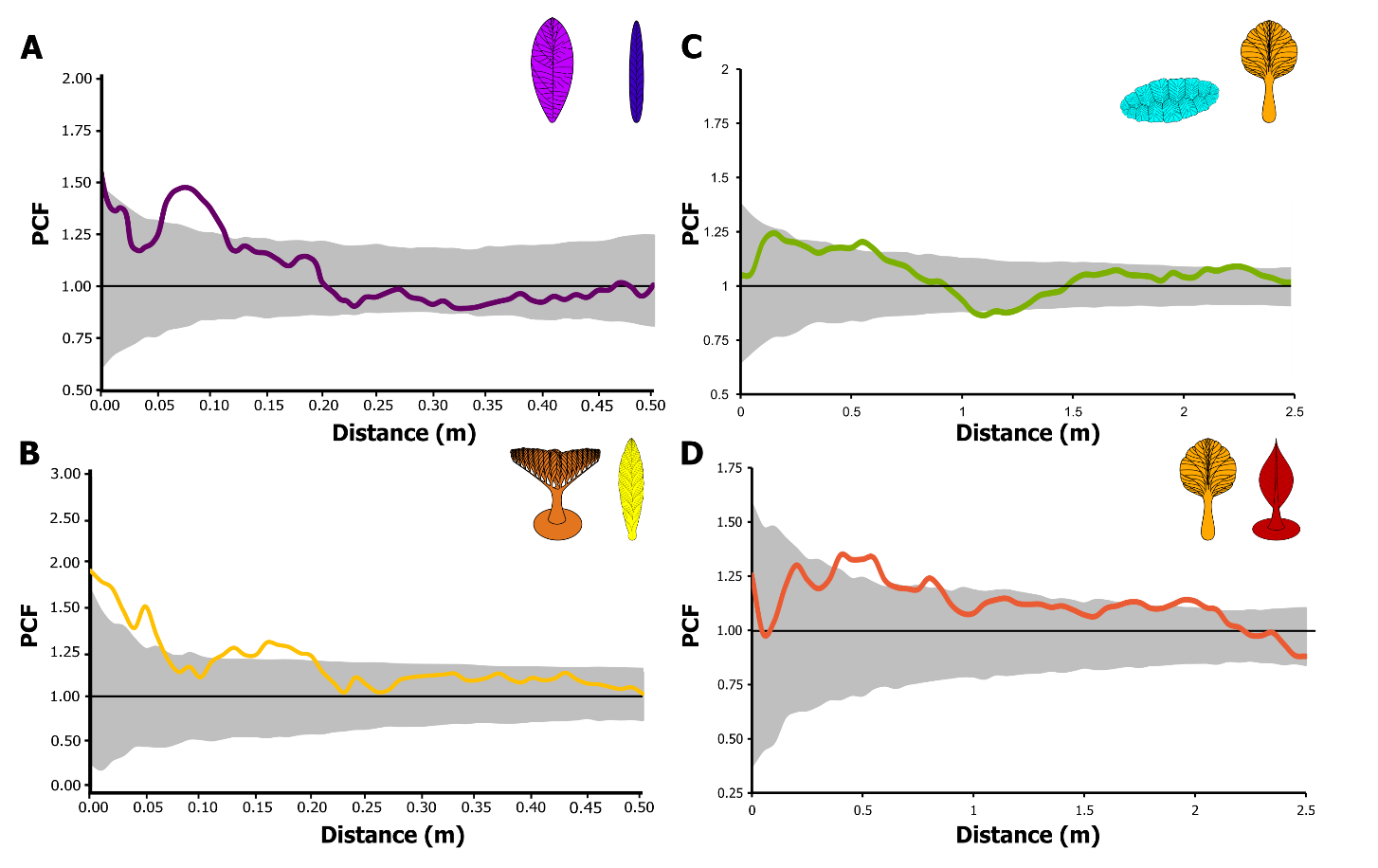


Figure S4. Toroidal Shift Bivariate PCF for the taxa which had non-independent spatial distributions. The grey area is the simulation envelope for 999 Monte Carlo simulations of the Toroidal Shift model. The x-axis is the inter-point distance between organisms in metres. On the y-axis, PCF=1 indicates complete spatial randomness (CSR) and is indicated by a black line, <1 indicates segregation, and >1 indicates aggregation. A) *Trepassia* and *Beothukis* from Spaniard’s Bay. B) *Charnia* and *Primocandelabrum* from Bed B, Charnwood Forest. C) Feather Dusters and *Fractofusus* and D) Feather Dusters and *Charniodiscus* from Mistaken Point ‘E’ Surface.

Supplementary Discussion

Small population spatial-distributions

Small populations (n<~70) run the risk of Type II errors when assessing model fit using Monte Carlo simulations due to wide simulation envelopes that scale with 1/n (Wiegand & Moloney 2013). To test the effect that Type II errors due to small populations may have on our analyses, we also tested model-fit of non-CSR models (HP, TC, DTC, ITC) using goodness-of-fit tests (Table 2) for those populations which exhibited CSR (as tested via Monte Carlo simulations). Of the nine CSR distributions found, three had a best-fit model of CSR (Fractofusus at Bristy Cove and Bradgatia on the ‘D’ and ‘E’ surfaces**)**, while the other six have a best-fit model of TC (Table 2). Therefore, while it is not correct to conclude that these six distributions are non-CSR, it is likely that if more data had been available, they would have been found to be non-CSR. These results do not change the overall result of neutral dominated communities because TC models (like CSR) describe neutral processes.

*Erosional analyses*

The present study found that there are erosional biases on the H14 surface, in contrast to previous work (Mitchell et al. 2015) that did not find any significant variation in specimen density across H14. The previous studied area is shown imposed on the H14 map used in this study (Fig. S2). Here we have mapped a larger area (82.4m^2^ versus 26.7m^2^), and so these analyses have been able to detect both signals over larger areas, and more subtle patterns. It’s therefore reasonable to conclude that the apparent discrepancy results from the difference in study area size.

*Spaniard’s Bay Surface*

Spaniard’s Bay is the only studied community that we found to be dominated by niche processes. We note that it likely records an immature community due to a limited size-range of small specimens (Brasier et al. 2013). Its unusual taphonomic regime, involving rapidly flowing currents that likely removed multiple specimens during burial (Brasier et al. 2013), prevents reliable determination of the extent to which the observed niche patterns on this surface reflect the original community assembly. The studied community at Spaniard’s Bay is relative small (59 specimens), so abundant species were defined as those with >15 specimens. We used this limit because the spatial distributions on this surface were sufficiently strong and non-noisy that they were sufficient for these analyses.

**
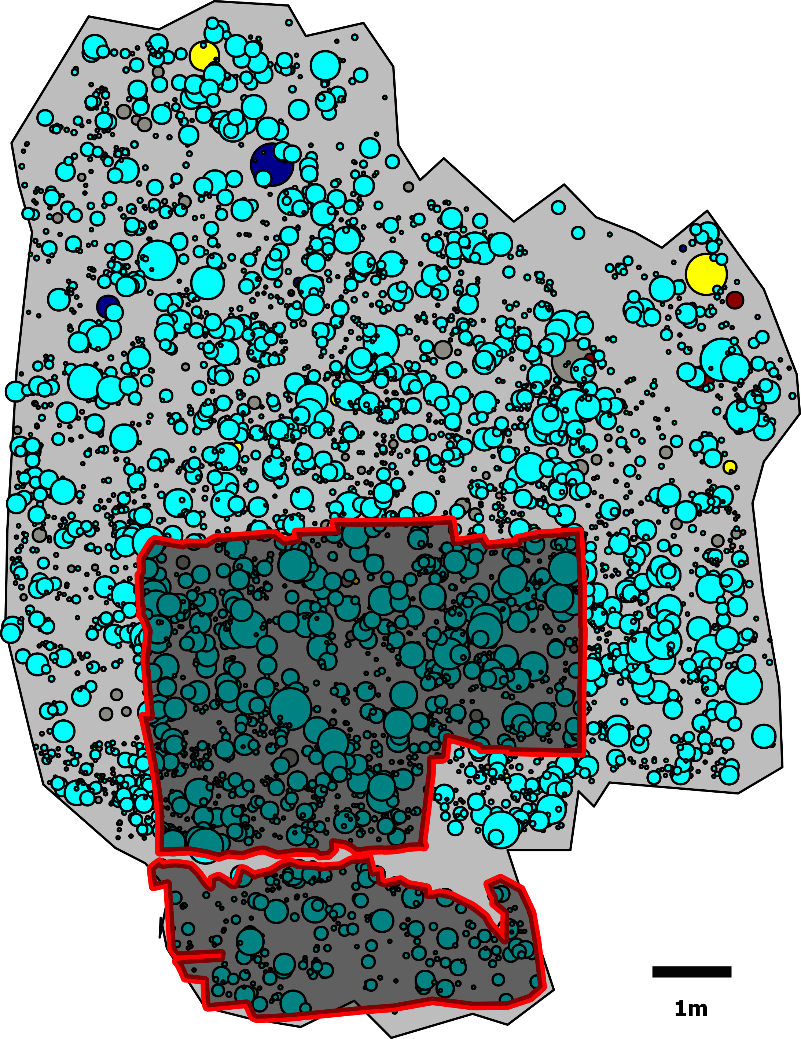
**

**Figure S5:** H14 spatial map showing the area mapped for Mitchell et al. 2015 study (red outline). Spatial maps show the positions of the fossil specimens, with the size of the circle indicated the length of the fossils (*Fractofusus*) or height (fronds) (indicated by a circle). Black scale bar = 1 m. Different colors indicate different taxa as follows: *Thectardis* navy; *Fractofusus* light blue; *Charnia* bright yellow; *Charniodiscus* dark red; Ivesheadiomorphs dark grey.

**Supplementary References**

Brasier, M. D., Liu, A. G., Menon, L., Matthews, J. J., McIlroy, D., & Wacey, D. (2013). Explaining the exceptional preservation of Ediacaran rangeomorphs from Spaniard's Bay, Newfoundland: a hydraulic model. *Precambrian Research*, 231: 122-135.

Mitchell, E. G., Kenchington, C. G., Liu, A. G., Matthews, J. J., & Butterfield, N. J. (2015). Reconstructing the reproductive mode of an Ediacaran macro-organism. *Nature*, 524: 343-346.
